# Benign and Infected Sacrococcygeal Teratoma in a 13-year-old Girl

**Published:** 2015-05-01

**Authors:** Kamal Nain Rattan, Kapil Bhalla, Jasbir Singh, Hemant Rattan

**Affiliations:** 1 Department of Pediatric Surgery, PGIMS, Rohtak, Haryana, India; 2 Department of Pediatrics, PGIMS, Rohtak, Haryana, India; 3 PDM Dental College, Bahadurgarh, Haryana, India

**Dear Sir,**

Sacrococcygeal teratoma (SCT) is usually found in the newborn period and now frequently being diagnosed prenatally on fetal ultrasound. Majority of the cases are found during the first few days of life, with less than 10% being diagnosed beyond the age of two year. Females are ten times more likely to be affected then males.[1] We herein report a case of benign and infected sacrococcygeal teratoma in a 13-year-old girl.

Patient presented with complaints of fever for five days and swelling in sacrococcygeal region. The swelling was present since birth. There was history of occasional urinary retention. She had no significant change in bowel habits. She was a student of sixth grade. She was born at home with uneventful antenatal and postnatal period. On examination, she had stable vital signs, pallor, febrile, bilateral chest was clear. Abdominal examination was normal. On local examination, there was a firm, tender and irregular mass of 10cm -12 cm in size in the sacral region (Fig.1). On per rectal examination a tender mass was palpable extending into presacral space, and finger could be insinuated above the swelling. Laboratory investigations showed hemoglobin level 8 g/dl. Rest of the tests were within normal limits. X-ray pelvis showed calcification. On ultrasonography of abdomen and pelvis, there was a mass of mixed echogenicity with cystic lesions in presacral space. CECT scan abdomen showed a multicystic lesion in pre-sacrococcygeal region containing foci of calcification with posterior extension up to skin (Fig. 2). On the basis of clinical and radiology diagnosis of sacrococcygeal teratoma type II was made. After preoperative optimization the patient underwent surgical excision of tumor in toto in prone position through perineal approach. Tumor contained pus (which was drained), along with cystic and solid elements. Post operative period was uneventful. Histopathological examination confirmed the diagnosis of mature sacrococcygeal teratoma. Postoperative alpha fetoprotein and ultrasound examination were normal. The patient is doing fine on follow-up.

**Figure:**
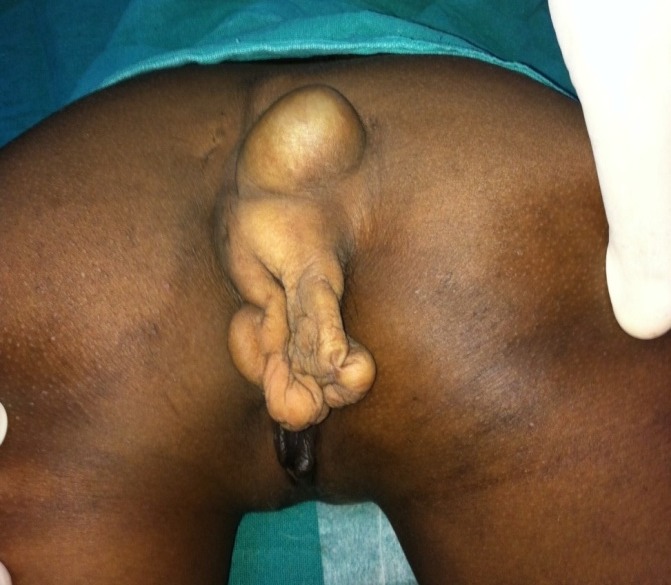
Figure 1: Large, irregular mass in sacral and perineal region.

**Figure:**
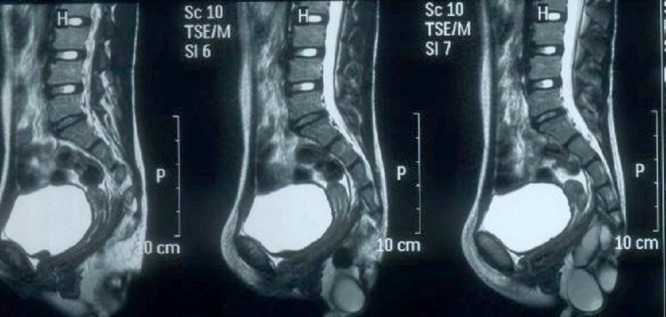
Figure 2: CECT Abdomen and pelvis showing large mass with calcifications in pre-sacral and perineal region.

SCT is the most common congenital tumor occuring in 1 in 35000 live births. Teratomas are classified as mature, immature, and malignant depending upon histopathological findings.[2,3] The propensity of malignant changes increases with age. Approximately 8% of SCTs are malignant at birth; by the age of 6 months, 40 % to 80% are malignant.[4] Presence of yolk sac tumor or other malignant component is main reason of malignant transformation of a SCT. It can be speculated in our case that the absenec of these malignant components may be a reason of non transformation to malignancy. Prognosis is excellent for mature teratoma, but malignant cases have a tendency to recur and metastasize. Regular follow up is required to detect early recurrence. We are following up our patient for last two years and found no recurrence.

## Footnotes

**Source of Support:** Nil

**Conflict of Interest:** None declared

